# Microbiological Safety and Presence of Major Mycotoxins in Animal Feed for Laboratory Animals in a Developing Country: The Case of Costa Rica

**DOI:** 10.3390/ani11082389

**Published:** 2021-08-13

**Authors:** Fabio Granados-Chinchilla, Carol Valenzuela-Martínez, Berny García-Murillo, David Aguilar-Madrigal, Mauricio Redondo-Solano, Andrea Molina

**Affiliations:** 1 Independent Researcher, Suite 23C, C115A Street, Curridabat, San José 11801, Costa Rica; fabio.granados@ucr.ac.cr; 2 Centro de Investigación en Enfermedades Tropicales (CIET), Sección de Microbiología de Alimentos, Departamento de Microbiología e Inmunología, Facultad de Microbiología, Universidad de Costa Rica, San José 11501-2060, Costa Rica; carol.valenzuela@ucr.ac.cr (C.V.-M.); bernygarcia25@gmail.com (B.G.-M.); mauricio.redondosolano@ucr.ac.cr (M.R.-S.); 3 Centro de Investigación en Nutrición Animal (CINA), Facultad de Microbiología, Universidad de Costa Rica, Ciudad Universitaria Rodrigo Facio, San José 11501-2060, Costa Rica; david2696@gmail.com; 4 Centro de Investigación en Nutrición Animal (CINA), Escuela de Zootecnia, Universidad de Costa Rica, Ciudad Universitaria Rodrigo Facio, San José 11501-2060, Costa Rica

**Keywords:** laboratory animals, murine models, animal feed, feed microbiological safety, *Salmonella*, indicator organisms, mycotoxins

## Abstract

**Simple Summary:**

The microbiological safety and quality of commercial animal feed for laboratory animals, produced in Costa Rica, was assessed. Analysis of the animal feed included general microbial markers (total coliforms and molds) and the behavior over time of two specific feed contaminants (*Salmonella* spp. and mycotoxins). Results from the study suggest that there is a low risk of contamination from viable microorganisms but the product contains important levels of mycotoxins. Current preventive measures (UV light disinfection) are not effective and additional handling protocols should be considered.

**Abstract:**

Safety and quality of compound feed for experimental animals in Costa Rica is unknown. Some contaminants, such as *Salmonella* spp. and mycotoxins, could elicit confounding effects in laboratory animals used for biomedical research. In this study, different batches of extruded animal feed, intended for laboratory rodents in Costa Rica, were analyzed to determine mycotoxin and microbiological contamination (i.e., *Salmonella* spp., *Escherichia coli*, total coliform bacteria, and total yeast and molds enumeration). Two methods for *Salmonella* decontamination (UV light and thermal treatment) were assessed. Only *n* = 2 of the samples were negative (representing 12.50%) for the 26 mycotoxins tested. Enniatins and fumonisins were among the most frequent toxins found (with *n* = 4^+^ hits), but the level of contamination and the type of mycotoxins depended on the supplier. None of the indicator microorganisms, nor *Salmonella*, were found in any of the tested batches, and no mold contamination, nor *Salmonella* growth, occurs during storage (i.e., 2–6 months under laboratory conditions). However, mycotoxins, such as enniatins and fumonisins tend to decrease after the fourth month of storage, and *Salmonella* exhibited a lifespan of 64 days at 17 °C even in the presence of UV light. The D-values for *Salmonella* were between 65.58 ± 2.95 (65 °C) and 6.21 ± 0.11 (80 °C) min, and the thermal destruction time (z-value) was calculated at 15.62 °C. Results from this study suggest that laboratory rodents may be at risk of contamination from animal feed that could significantly affect the outcomes of biomedical experiments. Thus, improved quality controls and handling protocols for the product are suggested.

## 1. Introduction

The quality and safety control of the diets for laboratory animals is crucial for the welfare of the animals and to ensure that experimental outcomes are not biased by unintended nutritional or contamination factors [1,2]. Chemical and biological contaminants of feeds, such as bacteria, bacterial toxins, and mycotoxins could influence toxicological, physiological, immunological, reproductive, and other types of research experiments [2,3]. In fact, animal feed, or the raw materials used for its fabrication, are prone to contamination, which can occur at any stage of processing or during storage [4].

Similar to pet food, rodent feed belongs to the group of foods called “foods with low moisture content,” whose main characteristic is the low *a_w_* (values below 0.85) [5]; it consists primarily of cereals, such as wheat, maize, barley, and other legumes such as soybean. Additionally, in Costa Rica, this type of feed usually contains animal (poultry, beef, and pork) by-product meals, which have shown a high prevalence of *Salmonella* contamination [6,7]. This is important, as foods with reduced *a_w_* levels are believed to be less permissive for microbial growth, but some pathogens can survive for extended periods under these conditions [8]. 

Bacterial infections of laboratory animals (both clinical and subclinical) can lead to abnormal responses to experimental treatments and interfere with research outcomes [9,10]. *Salmonella* spp. is a foodborne pathogen highly related to the contamination and survival in foods with low *a_w_* content [6,11]; it can survive under harsh conditions for extended periods, even when food products are subjected to high temperatures [12]. In fact, *Salmonella* recontamination of animal feed, after high-temperature treatment, has been reported [5,13] and, as a consequence, pet food has been documented as one of the leading causes of salmonellosis in humans and animals [14,15]. For example, in Canada, a total *Salmonella* prevalence of 12.5% was reported in pet food analyzed between 2002 and 2009 [14]. Based on this information, it can be hypothesized that *Salmonella* contamination could pose a problem in animal feed for laboratory purposes, as these bacteria can affect mice similarly to humans. Though scarce, salmonellosis reports in laboratory animals exist [9,16,17,18], and even zoonotic spread of the infectious disease has been reported [19].

Animal feed is also vulnerable to mycotoxin contamination in the field (in the case of raw materials) or during storage and this depends on pre- and post-harvest edaphoclimatic and environmental conditions [20]. Some factors, such as improper processing, packaging, drying techniques, and transport activities may also influence fungal growth and increase the risk of mycotoxin production [21]. Mycotoxins have also demonstrated to be refractory toward processing operations [22], meaning that they could be present in the final product used to feed laboratory animals. Local feed surveillance has already shown a considerable prevalence of mycotoxins in raw ingredients and compound feed [23,24]. Specifically, for rodent feed, considerable concentrations of deoxynivalenol (DON), nivalenol (NIV), ochratoxin (OTA), and zearalenone (ZEA) have been found in commercial feed samples [25]. More recently, Escrivá and coworkers found multiple toxins, including enniatin B, B_1_, and ZEA [26]; all these compounds may exert additional stress on laboratory animals, and results of scientific studies could be biased, thus leading to wrong conclusions. For example, ZEA has a demonstrated immunomodulatory effect in murine models [27]. The importance of mycotoxin contamination of animal feed is increasing and recent improvements in analytical methodologies to determine and confirm several toxins simultaneously in animal feed is the proof of that [28]. Considering these data, it is clear that the quality control of food, for laboratory animals, is becoming more critical to ensure that reliable in vivo results could be obtained.

Few institutions in Costa Rica house animals for laboratory purposes and scientific experiments. One example is the Laboratory of Biological Assays (LEBi) at the University of Costa Rica, whose main objective is to provide healthy animals for the purpose of scientific studies. To fulfill its objective, the animal feed used by LEBi must comply with the highest standards in terms of food quality and safety results, so experiments performed using these animals, are repeatable and scientifically sane [29]. However, given the low demand for experimental animals locally, the internal market of feed that complies with the requirements for scientific experiments is too small, and just two local producers and suppliers are in charge of providing the product. These suppliers, whose main activity is the processing of pet food, do not provide the microbiological data, if any, that they could generate internally in their facility, supporting the absence of pathogenic bacteria, quality, and indicator microorganisms. In addition, it has been noted that there could be inadequate management of the inventory of the product, as the batches are stored for long periods, which may increase the risk of contamination in the facility.

Rodent feed manufacturers do not provide information on their product safety controls and official controls in this matrix are scarce, as surveillance programs are mainly focused on feed for productive animals. Hence, in general, the food safety of the product used to feed these animals in Costa Rica is ignored. For this reason, the vivarium takes extra measures to ensure the quality of the product is acceptable for feeding the rodents. Some of these measures include a basic microbiological analysis (coliforms and total aerobic counts) and the exposition of the animal feed to UV light during storage to eliminate or reduce potential microbiological contamination. However, validation studies, to know the efficacy of this treatment, have not been conducted, and no information is available regarding other contaminants such as pathogens, toxins, or chemicals. As the safety characterization of the animal feed used at vivariums may not be thorough due to the absence of regular surveillance programs that include mycotoxins and *Salmonella* analysis, the real risk for the experimental animals is unknown, and potential corrective actions may not be in place.

The objectives of this study were (i) To generate information on the microbiological risk profile of the animal feed for laboratory animals used at LEBi through the analysis of relevant microorganisms (*Salmonella*, *Escherichia coli*, total coliform bacteria, and total yeast and molds) and mycotoxins. (ii) To raise awareness about this potential risk for biomedical research in developing countries with similar facilities. (iii) To evaluate the efficacy of current intervention methods for the product (UV light) and alternative procedures (thermal treatment) in case contamination occurs. The information was used to design recommendations for the management of microbiological risks on the feed used for experimental animals in settings with limited resources.

## 2. Materials and Methods

### 2.1. Sampling and Study Objects

In this study, different batches of extruded/pelletized animal feed intended for laboratory rodents fabricated in Costa Rica were sampled and analyzed. Feed samples are routinely stored in stacked 1–3 mm thickness polypropylene woven 30 kg sack bags and destined for animals prepared for biological testing by the Laboratory for Biological Essays (LEBi) at the University of Costa Rica (San José, Costa Rica).

#### 2.1.1. Sampling for Mycotoxin Analyses

For mycotoxin analysis, *n* = 16 different rodents extruded feed batches were sampled. The samples were acquired from two different local suppliers (*n* = 9 and *n* = 7 different feed batches from suppliers 1 and 2, respectively).

Mycotoxin sampling was performed according to the parameters underlined by the Feed Inspectors Manual [30]. To improve sampling representability and compensate for possible contamination hotspots, compound 5 kg samples from each batch of feed were obtained by collecting *n* = 50 increments of 100 g each. Additionally, the 5 kg sample was quartered, and a subsample milled using an mm sieve (Retsch ZM 200, Haan, Germany).

#### 2.1.2. Sampling for Microbiological Analysis

For *Salmonella* analysis, two representative samples per batch from *n* = 12 different batches were taken for feed dispatched from supplier 1. To analyze *Escherichia coli*, total coliform bacteria, enumeration of yeast, and molds *n* = 16 different feed batches were sampled from supplier 1 and 2. The sampling method, described in the previous section, was adjusted to be performed aseptically, so the same samples could be used for microbiological analysis. Hence, corers and spatulas were previously autoclaved. Microbiological analysis subsamples were recovered before preparation for toxin analysis.

### 2.2. Safety and Quality in Animal Feed

#### 2.2.1. Mycotoxin Analysis

The assays were performed using a multitoxin targeted MS-based LC approach (*n* = 26 toxins). Briefly, 25 g of each sample was extracted using 100 mL of an ACN:H_2_O:CH_3_CO_2_H solution (74:25:1). The mixture was then dispersed for 2 min using an Ultra-turrax^®^ homogenizer set at 18,000 rpm (T25, IKA, Werke GmbH & Co. KG, Staufen in Breisgau, Germany). The supernatant was recovered and then gravity-filtered through Whatman^®^ 541 ashless filters (GE Health Life Sciences Little Chalfont, Buckinghamshire, UK). A 2 mL aliquot was pipetted to a 25 mL volumetric flask its volume made-up with phosphate buffer solution (PBS, containing NaCl, 150 mmol L^−1^; KCl, 2.50 mmol L^−1^; Na_2_HPO_4_, 4.50 mmol L^−1^; KH_2_PO_4_, 1.50 mmol L^−1^ and adjusted at a final pH of 7.4). During sample cleanup, solid phase extraction cartridges were activated and conditioned with 2 mL MeOH and equilibrated with 2 mL of a MeOH/H_2_O solution (5:95 volume ratio, Oasis^®^ HLB columns, WAT094226, 3 cc, 60 mg, 30 μm particle size, Waters Corporation, Milford, MA, USA). Solvents and sample extracts were transferred through the columns at a maximum flow rate of 1 mL min^−1^ with the aid of a an SPE 12 port vacuum manifold (operating at 15 mmHg, 57.044, Visiprep™, Supelco Inc., Bellefonte, PA, USA). To recover the analytes, columns were washed with a 2 mL MeOH/H_2_O (5:95) solution, and 2 mL of MeOH was used to elute analytes. The resulting eluates were concentrated to dryness under vacuum at 60 °C (Centrivap, LABCONCO, Kansas City, MO, USA), reconstituted with 300 μL of MeOH, and transferred to HPLC polypropylene vial insert (300 μL, polymer feet, 5182–0549, Agilent Technologies, Santa Clara, CA, USA).

The analytical determinations were accomplished using chromatographic system equipped with a 1260 infinity quaternary pump (61311C), a column compartment (kept at 40 °C during analysis, G1316A), and an automatic liquid sampler module (injection volume set at 10 μL, ALS, G7129A), and equipped with Zorbax Eclipse Plus chromatographic column (3.0 mm ID × 100 mm, 3.5 μm, P/N 959961–302, Agilent Technologies). The apparatus was coupled with a single quadrupole mass spectrometer with electrospray ionization ion source (6120B, Agilent Technologies). The drying gas, nebulizer pressure, drying gas temperature, and capillary voltage were set to 10.0 L min^−1^, 50 psi, 350 °C, and 4000 V, respectively, for positive ion mode electrospray ionization (ESI^+^).

A gradient analysis based on A ACN and B H_2_O as solvents, both acidified with formic acid at 0.1 mL/100 mL, was employed to separate the mycotoxins quantitatively at a flow rate of 0.15 mL min^−1^. The established gradient was as follows: at 0 min 10% A, at 4 min 10% A, at 22 min 100% A, at 25 min 10% A, and finally at 35 min 10% A. Toxins were assessed using a Selected Ion Monitoring/SIM mode, peak width and cycle time were set to 0.1 min and 0.60 s cycle^−1^, respectively. The molar mass, target ions, retention times, cone voltage, and obtained limits of detection and quantification of each analyte were described previously [23]. Finally, a naturally contaminated cornmeal (TR-MT100, Multitoxin Reference Material MT-C-999-G, Trilogy^®^, Washington, MO, USA) was tested parallel for quality control purposes during each batch of analysis.

#### 2.2.2. Water Activity (*a_w_*)

As previously described [31], the Aqualab^®^ chilled mirror technique was used to determine water activity in the samples (Aqualab^®^ 4TE, Metter Food, Pullman, WA, USA). A small portion of ca. 1 g of sieved material was placed in plastic cups and placed inside the chamber until equilibrium was reached (2.5 min on average). The *a_w_* values were registered with ±0.003 accuracies at 24.50 ± 0.24 °C. Water activity was measured for each feed immediately after sampling and when attained and was also monitored for six months during storage. AAFCO check sample 201921 (Equine Feed) and a verification solution (0.500 *a_w_*, 8.57 mol kg^−1^ LiCl) were used for quality control during measurements.

#### 2.2.3. Microbiological Analysis

Samples were homogenized using a laboratory blender (Interscience^TM^ BagMixer^®^ 400, Saint-Nom-la-Bretèche, France). Subsequently, *Salmonella*, *Escherichia coli*, total coliform bacteria, and total yeast and molds enumeration were performed. 

The protocol to isolate *Salmonella* was performed according to the Bacteriological Analytical Manual (BAM) method: *Salmonella*, Chapter 5 (FDA). Suspicious colonies on solid media were confirmed with the VITEK 2 system (Biomérieux, Durham, NC, USA) following the manufacturer’s instructions.

For the quantification of *Salmonella*, the samples were homogenized with 90 mL of 0.1 g/100 mL Sterile Peptone Water (PW; Oxoid Ltd., Basingstoke, UK) using a stomacher blender; additional decimal dilutions were applied to this original suspension using PW-containing tubes, and a volume of 100 µL of proper dilutions were used to inoculate Tryptic Soy Agar (TSA; Oxoid Ltd., Basingstoke, UK) and Xylose Lysine Deoxycholate Agar (XLD; Oxoid Ltd., Basingstoke, UK) plates. Agar plates were incubated at 35 °C for 24 h, and typical *Salmonella* colonies were quantified to obtain the population from each sample (log CFU g^−1^).

Coliform bacteria were analyzed following APHA/CMMEF methods 9.91–9.94 based on an MPN technique.

The enumeration of yeast and molds was analyzed following the respective method of the Bacteriological Analytical Manual [32].

### 2.3. Contaminants Behavior in Animal Feed during Storage

#### 2.3.1. Mycotoxins and Molds Growth during Storage

A randomly elected sample from each batch of extruded animal feed and supplier [batches *n* = 8 and *n* = 1 from suppliers 1 (S1) and 2 (S2), respectively] was conserved under laboratory conditions [i.e., from 21.5 to 28.7 (mean 24.5) °C and 29.7 to 56.1 (mean 37.8) %RH, respectively] and monitored for a_w_, mycotoxin and molds values for several months for a total of *n* = 9 replicate per sample (one per month) were taken over time to see the behavior of both batches.

#### 2.3.2. Bacterial Strains for Animal Feed Contamination

Five different *Salmonella* strains, including various serotypes (*S*. Typhi, *S*. Typhimurium, and *S*. Enteritidis), were used in this study. According to previous research in our laboratory, the selected strains demonstrated higher resistance to low water environments (not published). All the isolates are part of the bacterial collection of the Research Center for Tropical Diseases (CIET) from the University of Costa Rica (San José, Costa Rica), and they were maintained as glycerol stocks at −80 °C.

#### 2.3.3. *Salmonella* Growth in Animal Feed during Storage

Each *Salmonella* strain was grown in Tryptic Soy Broth (TSB; Oxoid Ltd., Basingstoke, UK) and incubated at 35 °C for 24 h (final *Salmonella* population of approximately 9.0 log CFU mL^−1^). Then, a suspension for each strain was prepared by making decimal dilutions in PW to obtain a final *Salmonella* population of around 6.0 log CFU mL^−1^. Equal volumes of each strain suspension were mixed to obtain a *Salmonella* cocktail used to inoculate 10 g of the animal feed by adding 100 µL (final population in the product 3.0–4.0 log CFU g^−1^) of the bacterial suspension. A small volume of bacterial suspension was used in order to avoid significant alterations of the original water activity of the samples. The inoculated samples were thoroughly mixed by hand inside a sterile plastic bag, and they were dried out inside a biosafety cabinet for 15 min to remove excess humidity and promote bacterial attachment. Then, each sample was sealed in plastic bags and stored at 17 °C inside the LEBi warehouse. At different time intervals, samples were taken from storage to quantify the *Salmonella* population on solid media. Three repetitions were performed to build growth curves.

#### 2.3.4. *Salmonella* Survival in Animal Feed during Storage and UV Resistance

For this experiment, the animal feed was inoculated as stated before but this time using the original *Salmonella* cocktail suspension with no dilutions (initial population in the sample between 6.0–7.0 log CFU g^−1^). Inoculated samples were stored at 17 °C inside the LEBi warehouse. A parallel set of samples was also prepared to validate the current UV radiation treatment on *Salmonella* present in animal feed; in this case, stored samples were exposed to continuous UV radiation (255 nm) using a UV lamp (Sylvania Germicide Lamp T8 30 W, San José, Costa Rica). Each sample bag was placed separately on a rack that was directly under the UV lamp (distance of 1.75 m). For both experiments, samples were removed from storage at different time intervals to quantify the *Salmonella* population as described (total sampling time of 64 days). Three repetitions were performed, and complete survival curves were constructed. A comparison was made between samples stored under normal conditions and those exposed to UV radiation.

The inoculated feeds were storage at LEBi warehouse under the same conditions that lab feeds are normally stored there, including a temperature of 17 °C. This temperature is used to avoid rancidity and improve shelf life, as natural-ingredient diets should be stored at temperatures less than 21 °C (ca. 70 °F, and a RH of 50%) [1]. Under these conditions, dry laboratory animal diets stored properly can be used up to 6 months, or longer in some cases, after manufacture [1].

### 2.4. Salmonella Thermal Resistance in Animal Feed

As described before, animal feed samples were prepared and inoculated with the *Salmonella* cocktail (initial *Salmonella* population of 7.0 log CFU g^−1^). The inoculated animal feed samples (10 g) were packaged in sterile plastic bags and immersed in a water bath with circulation capabilities (Isotemp 3013H, Thermo Fisher Scientific, Waltham, MA, USA) that was already set to the desired temperature. The time required to reach the target temperature (come-up time) was determined using non-inoculated product.

The different time-temperature combinations used for this experiment can be seen in Table 1. After heating at the specific time, samples were removed and immersed in an ice-water bath. Then, *Salmonella* population after heating was determined by quantification on solid media as described before. Data was used to construct thermal death curves to calculate D-values with the inverse of the slope of the regression line using Excel software (2007, Microsoft, Redmond, WA); the D-values are expressed in minutes or hours. Similarly, the thermal destruction time (z-value) was calculated by plotting the temperature (*x*-axis) versus log D-value, and the data was also fitted by linear regression (2007, Microsoft).

### 2.5. Statistical Analysis 

#### 2.5.1. For Mycotoxins Analysis

Spearman rank-order correlation tests were applied to determine the association between variables sampling date and *a_w_* between feed batches, *a_w_* and FB_1_ and FB_2_ concentration in a single sample over time. Simpson (*D*) and Shannon’s (*H*) Diversity Indexes were used to assess variation on mycotoxins among samples from different batches [33]. A *t*-student test was used to compare concentration means for the toxins shared between the supplier’s samples (i.e., ENNB, ENNB_1_, FB_1_, FB_2_, β-ZON) to assess whether they differed statistically. A similar test was used to determine if *a_w_* values differed among suppliers. For all statistical tests, a threshold value of α = 0.05 was used to consider differences between variables significant. 

#### 2.5.2. For *Salmonella* Behavior Analysis

As represented by animal feed batch, *Salmonella* stock, and day of preparation, three independent replications were performed for each of the experiments. Data (log CFU per gram, D-values, and *z*-values) were compared using analysis of variance of the General Linear Model procedure of the Statistical Analysis System (SAS Institute Inc., Cary, NC, USA). Fisher’s least significant difference (*p* < 0.05) was used to separate the means.

## 3. Results

### 3.1. Water Activity between Different Feed Batches

Table 2 shows the water activity analysis for the samples and batches analyzed from suppliers 1 and 2. These values confirm that most samples from both suppliers can be classified as dry food (i.e., *a_w_* values < 0.60). Suppliers 1 and 2 showed *n* = 2 and *n* = 1 samples with *a_w_* values above 0.60, which would classify them as intermediate moisture foods. Finally, there does not appear to be a trend regarding the sampled batch of feed and its water activity (Spearman rho, *p* = 0.399).

### 3.2. Safety and Quality of Animal Feed

#### 3.2.1. Mycotoxins of Animal Feed

During the study, of the total samples analyzed (*n* = 16), only *n* = 2 of the samples were negative (representing 12.50%) for the 26 analytes tested. Both negative samples listed above correspond to supplier 1 (samples S1C and S1D).

**Figure 1:**
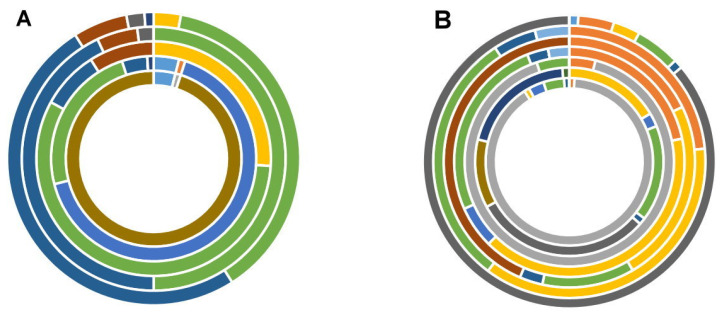
Distribution of mycotoxins for (**A**) (*n* = 9 samples of laboratory animal feed for supplier 1). Key (from inner to outer ring): Feed batch S1A. 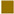 NIV (95%), 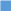 15-ADON(4%), 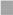 AFB_1_ (1%). Feed batch S1F. 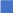 DON (66%), 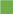 ENNB (24%), 15-ADON (4%), 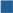 ENNB_1_ (4%), 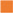 3-ADON (1%), 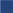 ZEA (1 %). Feed batch S1G. ENNB (57%), 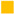 β-ZON (26%), 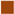 FB_1_ (9%), ENNB_1_ (8%). Feed batch S1H. ENNB (50%), ENNB_1_ (43%), FB_1_ (5%), 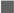 FB_2_ (2%). Feed batch S1I. ENNB_1_ (50%), ENNB (38%), FB_1_ (6%), β-ZON (3%), FB_2_ (2%), ZEA (1%) and (**B**) (*n* = 7 samples for supplier 2). Key (from inner to outer ring): Feed batch S2A. 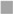 DON (90%), 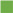 FB_1_ (4%), 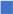 ENNB_1_ (3%), 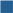 FB_2_ (1%), 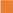 β-ZON (1%), 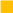 ENNB (1%). Feed batch S2B. 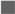 HT-2 (26%), 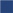 OTA (17%), FB_1_ (14%), ENNB (14%), 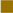 NEO (10%), ENNB_1_ (2%), FB_2_ (1%), 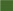 STE (1%) Feed batch S2C. DON (90%), FB_1_ (5%), β-ZON (4%). Feed batch S2D. ENNB (41%), FB_1_ (26%), β-ZON (22%), ENNB_1_ (6%), FB_2_ (3%), 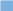 ZEA (3%). Feed batch S2E. 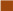 FSX (44%), ENNB (24%), β-ZON (18%), FB_1_ (12%), FB_2_ (3%). Feed batch S2F. ENNB (36%), FB_1_ (30%), β-ZON (23%), FB_2_ (5%), ZEA (4%) G. HT-2 (87%), FB_1_ (5%), β-ZON (4%), ENNB (3%), 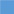 3-ADON (1%), FB_2_ (1%). Each ring represents a different batch of rodent feed tested. Values within brackets are relative to the total concentration found for each sample (toxins < 1% not shown).

Regarding the rest of the samples analyzed from supplier 1, for *n* = 2 samples (S1B and S1E), only *n* = 1 toxin was observed, i.e., enniatin B_1_ and aflatoxin B_1_ with concentrations of (143.11 ± 17.49) μg kg^−1^ and (0.15 ± 0.02) μg kg^−1^, respectively (Table 3). The remaining samples exhibited *n* = 3 (11.54%), *n* = 9 (34.62%), *n* = 5 (19.23%), and *n* = 7/26 (26.92%) analytes respectively for samples S1A, S1F, S1G, S1H, and S1I (Figure 1A). For supplier 2, of the *n* = 7 samples tested from their food for laboratory animals, all of the samples exhibited a mixture of at least five or more toxins (Figure 1B).

Unlike samples from supplier 1 (*D* = 8.67, *H* = 2.27), the toxins present in supplier 2′s feed are more varied (*D* = 12.47, *H* = 3.44). Enniatins were predominant in samples from supplier 1, but any toxin was in higher frequencies in supplier 2’s feeds (Figure 1A,B, Table 3). Enniatins and fumonisins are among the most frequent toxins found (with *n* = 4^+^ hits), and the samples from both suppliers share this trait. Average concentrations for both suppliers 1 and 2 are shown in Table 3. For supplier 1, higher levels of mycotoxin were observed for ENNB, ENNB_1_, and 15-ADON; for supplier 2, FB_1_ was present at higher values. For supplier 1 and supplier 2 samples, AFB_1_, and β-ZON were frequently found with concentrations of 3.30 ± 5.38 and 268.71 ± 527.85 μg kg^−1^, respectively on both accounts.

#### 3.2.2. Indicator Microorganisms and Pathogens in Animal Feed

The enumeration of yeast, molds, coliform bacteria, and *E. coli* were used as indicators of hygiene and fecal contamination of animal feed intended for laboratory animals. In all feed batches tested, none of the indicator microorganisms were found. Similarly, the analysis of the detection of *Salmonella* was used to assess the microbiological safety of animal feed. All feed samples tested were *Salmonella* negative.

### 3.3. Microbiological and Mycotoxin Analysis of Lab Animal Feed during Storage

#### 3.3.1. Mycotoxin, Water Activity and Mold Analysis on a Feed Batch over Time

**Figure 2:**
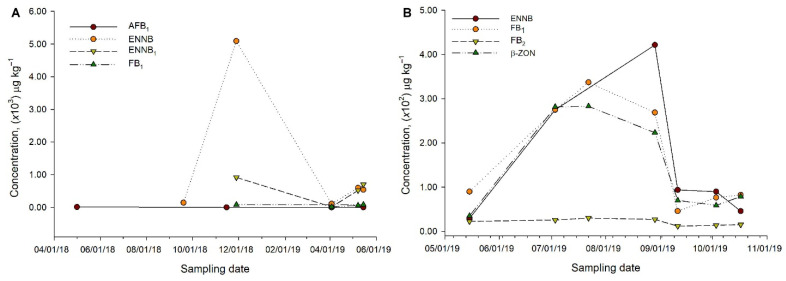
Analysis of the most relevant toxins during animal feed storage for (**A**) (supplier 1) and (**B**) (supplier 2).

There is no marked trend overtime on mycotoxin values in the samplings for supplier 1 (Figure 2A). However, for supplier 2, there is a notable trend for ENNB, FB_1_, and β-ZON, where higher concentrations were observed for the first three feed samplings; later, these concentrations decrease over time (Figure 2B).

For supplier 1, the last sample tested exhibits a higher number of toxins *n* = 6 (FB_1_, AFG_2_, FB_2_, AFB_1_, ENNB, ENNB_1_) compared to those found in the initial sampling *n* = 3 (FB_1_, FB_2_, β-ZON). A similar situation is observed for the follow-up of supplier 2, a reduction in the number of analytes is observed, this is *n* = 7 (FB_1_, AFG_2_, FB_2_, AFB_1_, β-ZON, ENNB, ENNB_1_) versus *n* = 3 (FB_1_, FB_2_, β-ZON), respectively (Table 4).

**Figure 3:**
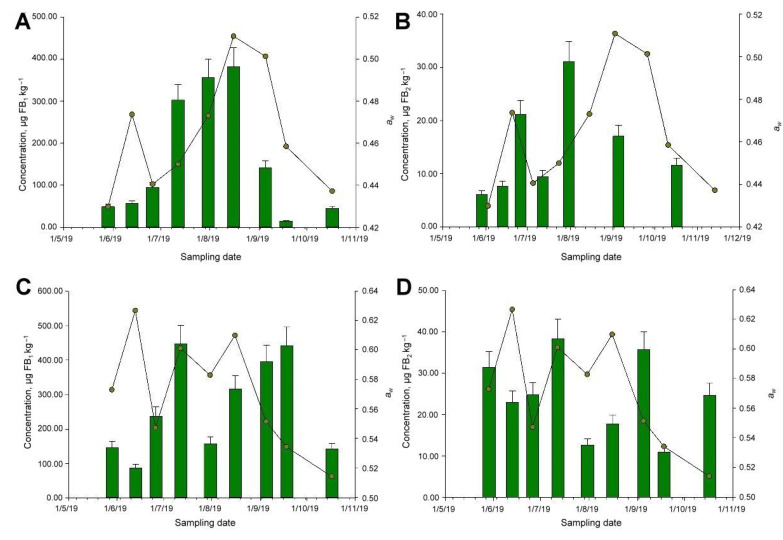
Behavior of fumonisin fractions B_1_ (**A**,**C**) and B_2_ (**B**,**D**) and of water activity over time for a stored animal feed Figure 1. (**A**,**B**) and supplier 2 (**C**,**D**). Green bars represent fumonisin levels, whereas line and scatter plots represent *a_w_* levels. Fumonisin B_1_ and B_2_ were selected, as they are the most common toxins found in the samples tested.

In general, no marked trend was observed for water activity or toxin levels (Figure 3B–D), except for the case of FB_1_ and the sample selected from supplier 1 (Figure 3A). However, there is no causality or association between variables (Spearman rho, *p* > 0.05). Finally, no mold contamination was detected during this storage period (i.e., <100 CFU g^−1^).

#### 3.3.2. *Salmonella* Behavior during Storage after Inoculation

##### Bacterial Growth

The five *Salmonella* spp. strain cocktail inoculated at low concentrations in the animal feed for rodents did not show any growth after 48 days of storage at 17 °C. The initial population in the animal feed was 3.4 ± 0.4 log CFU g^−1^ and after 48 days the final population was 1.7 ± 0.1 log CFU g^−1^ for a total decrease in 1.7 log CFU g^−1^.

##### Bacterial Survival

**Figure 4:**
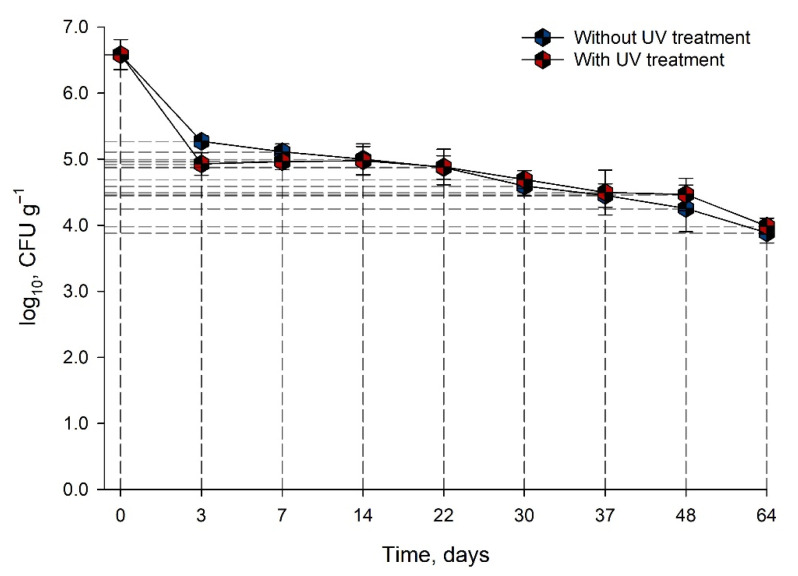
Survival behavior of *Salmonella* spp. in feed stored at 17 °C, with and without constant exposure to UV radiation.

*Salmonella* survived for 64 days in animal feed stored at 17 °C (Figure 4). The initial population of the *Salmonella* spp. cocktail was 6.6 ± 0.2 log CFU g^−1,^ and a final population of 3.9 ± 0.2 log CFU g^−1^ was observed after 64 days of storage at 17 °C for a total decrease in *Salmonella* spp. numbers of 2.7 ± 0.2 log CFU g^−1^. A significant decrease (*p* < 0.05) in *Salmonella* spp. population was observed between days 1 (6.6 ± 0.2 log CFU g^−1^) and 3 (5.27 ± 0.08 log CFU g^−1^). However, after day 3 of storage, no significant decrease in *Salmonella* population was observed, and the average reduction value obtained was just 0.04 log CFU g^−1^ per day. Similar results were observed in the case of samples stored under UV light (Figure 4) and no differences (*p* > 0.05) were determined between both conditions.

### 3.4. Salmonella Resistance in Animal Feed

#### *Salmonella* Thermal Resistance in Animal Feed

**Figure 5:**
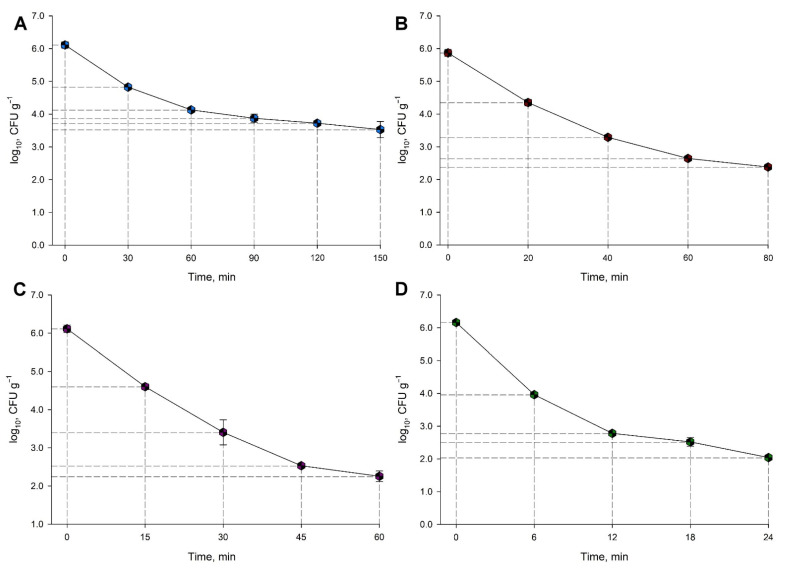
Thermal destruction curves of *Salmonella* spp. after inoculated feed was treated at 65 °C (**A**), 70 °C (**B**), 75 °C (**C**), and 80 °C (**D**).

Figure 5A–D show the thermal resistance of *Salmonella* spp. in animal feed at four different temperatures: 65 °C, 70 °C, 75 °C, and 80 °C. Survival and temperature share an inverse association with deltas of −2.58, −3.49, −3.86, and −4.11 log CFU g^−1^, for each condition, respectively. Time and temperature also share the same relationship with 150, 80, 60, and 24 min to reach a similar decrease in *Salmonella* population.

The D-value was calculated for each temperature (Table 5) using the straight-line equation, which is the negative inverse of the slope.

**Figure 6:**
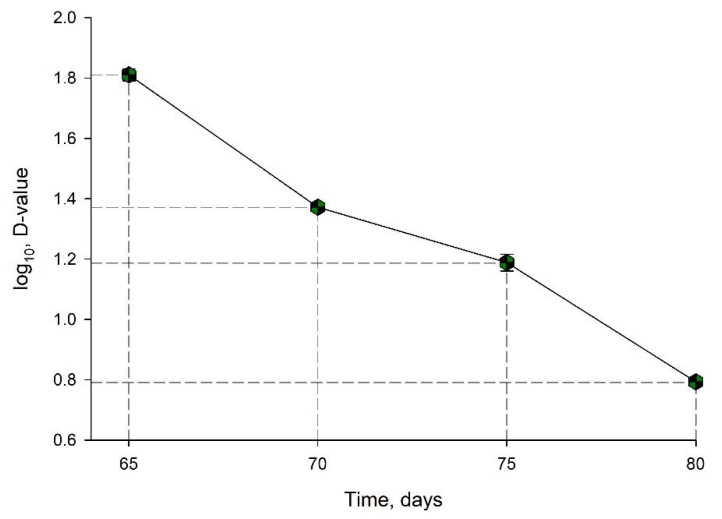
Thermoresistance curve for the mixture of *Salmonella* spp. in feed for rodents.

The log D-values for each temperature were used to build the thermoresistance curve of *Salmonella* in animal feed (Figure 6). Using the negative inverse of slope of the equation of the line (i.e., *y* = −0.1614 + 5.4303, *R* = 0.9275, standard error of estimate 0.7128) a z-value of 15.62 °C was calculated.

## 4. Discussion

### 4.1. Safety and Quality in Animal Feed

#### 4.1.1. Mycotoxin Analysis between Different Feed Batches

Mycotoxins are secondary metabolites produced by filamentous fungi belonging mainly to the genera *Aspergillus*, *Fusarium*, *Penicillium,* and *Alternaria* [34]. Simultaneous presence of several toxins in animal feed has been described as having significant adverse effects, even at low concentrations, given additive or synergistic interactions [35,36]. In this sense, the analysis of the co-occurrence of several toxins simultaneously in an animal feed is of utmost importance [36]. 

The co-occurrence of mycotoxins in animal feed has already been described for products intended for other species, which has already shown that it can exert antagonistic, additive, or synergistic effects on animals [36]. In this regard, data reported herein are in line with those reported elsewhere for rodent feed [i.e., 0.3 (OTA) to 298 (DON) and 6.4 (ENNA_1_) to 303.6 (ZEA) μg kg^−1^] [12,13]. Besides toxins, other environmental contaminants have been described in rodent feed (e.g., PCBs, heavy metals, PCDD/Fs, and pesticides [4]. This will exert additional metabolic stress on the animal, and in the presence of xenobiotics, mycotoxins could potentiate their injuriousness. 

In addition, technological processes on raw materials, or balanced foods, have proven to be ineffective in post-harvest control of these contaminants [35]. This is relevant since rodent feed is processed through an extrusion process (i.e., subjected to temperatures ca. 150 °C under high pressure when forced through the die) [1]. By consensus, it has been described that the best strategy for the post-harvest control of mycotoxins is based on proper storage and handling of animal feed to prevent conditions that lead to fungal growth [37]. Temperature, water activity, and the presence of insects have been cited as the factors most associated with the formation of mycotoxins during storage [37].

On the contrary, it is typical that the by-products of food materials used in human consumption (usually categorized in this context as waste) are reprocessed on occasion to be incorporated into the balanced animal feed. For example, rice husk is used as a vehicle to introduce balanced vitamin mixtures, but the outer part of this grain is the most prone, due to its exposure, to contamination by toxins [38]. Under these conditions, an 11.6% prevalence for aflatoxins in laboratory animal feeds has been reported for local products [38].

Although the microbiological tests did not show the presence of fungi or yeasts in any of the samples tested, it should be noted that the mycotoxin contamination could have occurred at any point prior to its extrusion [39], or it could be a consequence of raw materials already contaminated [40]. Furthermore, mycotoxins can remain in the finished product even without the presence of the fungus that synthesized them since they are molecules that are highly resistant to industrial unit operations [21]. The presence of trichothecenes, produced by *Fusarium* sp., neosolaniol and fusarenone-X, indicates the use of raw materials from temperate climates [41].

Mycotoxins, depending on the type, can cause different effects in animals usually used for experimentation, such as rats, mice, and rabbits. These effects include inhibition of the immune system, carcinogenic, teratogenic, hepatotoxic, nephrotoxic, endocrine and reproductive disorders, sterility, among others [24,25].

Herein, we reported especially high, and frequent, rodent feed contamination with enniatins and fumonisins. Similar trends have been detailed previously for other feedstuffs in Costa Rica, with prevalence of contamination reaching, in some cases, up to 45–50% and with non-trivial concentrations [23,24].

Specific adverse effects in vitro, and in vivo, have been reported for aflatoxin B_1_, enniatin, fumonisins, zearalenol-derivatives in animals, especially murine models. These effects include immunotoxicity, oxidative stress for AB_1_ and FB_1_ [42], and acute genotoxicity in hepatocytes for AFB_1_ [43,44]. Noteworthy, despite presence of AFG_2_, levels do not exceed the legal thresholds of 20 μg kg^−1^ [45,46].

FB_1_ and DON have been demonstrated to alter the intestinal barrier, impair the immune response, and reduce feed intake and weight gain. Their presence in feed increases the translocation of bacteria; mycotoxins can also enhance the susceptibility to infectious diseases [47]. Additionally, blastocyst cell numbers and proportion of late blastocysts have been reported to decrease in mouse embryos in the presence of T-2 and FB_1_ [48].

Beauvericin and both enniantins have demonstrated cytotoxicity of Caco-2 cells [49], neurotoxicity (can cross the brain-blood barrier, [50], genotoxicity [51], and intestinal toxicity) [52].

Finally, ZEA and derivatives have demonstrated toxic effects in mice’s reproductive system [53]. In granulosa, cells from mice and domestic animals have shown impaired development and follicle steroidogenesis, reduced oocyte nest breakdown, damaged meiotic progression, poor fetal oocyte survival, accelerated primordial follicle activation, and enhanced follicle atresia [54].

Data from this study indicate that laboratory animals could be chronically exposed to mycotoxin contamination from animal feed. Further studies are necessary to understand the clinical significance of this exposure, considering both the levels of mycotoxins reported in this study, the amount of feed that is consumed, and the regular lifespan of the rodents. However, considering a batch of food could last several weeks, or even months, a variation in the concentration of toxins during storage generates a more troublesome scenario by implying that some rodents would have a higher exposure to chemicals than others. The same situation arises if, for any reason, the feed were to be contaminated during storage.

#### 4.1.2. Microbiological Quality of Samples

In line with the local legislation thresholds for animal feed (i.e., absence in 25 g) [45,46], data from this study confirm that the animal feed for rodents was not contaminated with *Salmonella* spp. or any indicator bacteria. The animal feed is an extruded product where most of the pathogenic microorganisms in the raw material are eliminated during processing [55]. Despite a high risk of post-processing contamination of this kind of food [56], *Salmonella* spp. or any microbial indicator were not detected, meaning that proper storage and handling conditions are being applied. At least this could be concluded for those batches analyzed in this study. In addition, some chemical or biological antimicrobial compounds (preservatives and antibiotics) present in the product could inhibit the growth of *Salmonella* spp. Although antibiotics are not allowed in animal feed according to applicable regulation [45,46], antimicrobial residues in compound feeds have been reported previously [23,57]. Then, it is possible that animal feed used by LEBi may also contain unintended antimicrobial compounds residues.

On the other hand, it is important to clarify that the sub-samples used for the study are just a representation of larger product volumes. This means that only a small fraction of each batch was analyzed. It is possible that the presence of *Salmonella* could be underestimated because its distribution is not homogeneous within each bag of product, and it could have been present only in certain spots within small microenvironments [58]. Based on the experience at LEBi, it is challenging to obtain new batches every time the animal feed is bought. As LEBi requests only small amounts of animal feed, the food supplier may provide the same batch of animal feed for an extended period of time. This indicates that an exhaustive sampling analysis may be needed to discard the presence of *Salmonella* spp. 

Furthermore, indicator microorganisms, of fecal contamination or poor hygiene, were not found, which supports the effectiveness of the extrusion process and the adequate post-process handling. In addition, during storage, the growth of fungi and molds was not detected; this could be due to calcium propionate as an additive in the animal feed tested. Calcium propionate, in its acid form, is an inhibitor of fungal growth, causing the inactivation of enzymes. It also competes with essential amino acids, such as alanine, thus inhibiting microorganisms’ growth [59,60].

Given that post-processing contamination with *Salmonella* spp. risks do exist for heat-treated feed and feed ingredients (e.g., prevalence of 3.37 and 26.74% for Costa Rican pet food and meat and bone meal, respectively [6,7]), it could be assumed that the safety of the animal feed may be compromised at some point. However, the results from microbiological analysis, of the animal feed at LEBi, support the idea that there is a small risk of microbiological contamination for rodents. This means that chances of affecting the experimental animals (at least with viable microorganisms) are low, and the product may remain stable throughout storage. 

### 4.2. Water Activity between Different Feed Batches

It should be mentioned that *a_w_* values above 0.85 are considered a threshold to favor the growth of pathogenic bacteria [61]. On the other hand, fungi and yeasts are capable of growing with these values [61]. Thus, the *a_w_* values also justify the general absence of pathogens or counts below the lower limit, in all the samples, for indicators such as total coliforms and *Escherichia coli* (<3 CFU g^−1^) and fungi and yeasts (<100 CFU g^−1^).

### 4.3. Contaminants Behavior in Lab Animal Feed during Storage

#### 4.3.1. Water Activity and Toxin Behavior on a Feed Batch over Time

While at the end of the tests, both *a_w_* and mycotoxins present a downward trend, probably due to the effect that cold air has on the humidity of the product. Because the food is not hermetically sealed, there is gas transfer [62]. 

As in the case of *Salmonella*, it is possible to appreciate, in all cases, that sampling is essential to obtain accurate and accurate data [63]. There is inherent variability in the test as there are hot spots where the toxin may be since it is usually not distributed homogeneously in raw material or food [64]. Mycotoxin variation, within each sample, during storage might result from several factors, including abiotic and biotic-mediated hydrolysis of masked mycotoxins (e.g., glycated toxins) [65], microbial toxin metabolism [66], and toxin heterogeneity, within the feed, due to specific areas of elevated water activity [64]. 

Even though the feed samples tested can be considered microbiologically sound, based on general markers tested, it is relevant to remember that even feed samples that have been subjected to processing are not considered 100% sterile. Indeed, we have already reported *Staphylococcus*, *Bacillus*, or *Lysinibacillus* sp. in Costa Rican extruded feed samples [57].

#### 4.3.2. *Salmonella* Behavior during Storage

According to our results, *Salmonella* spp. does not have the ability to grow in animal feed during storage. It seems that the low water activity levels were a significant factor for preventing the bacteria from multiplying. These results agree with previous reports in the literature where it is shown that pathogenic bacteria such as *Salmonella* spp. are not able to grow in *a_w_* values below 0.85 [67]. Similarly to our study, Beuchat reported that *Salmonella* spp. did not have the ability to grow in inoculated pecans (1.53 log CFU g^−1^) that have *a_w_* values between 0.43 and 0.51 [68]. Although *Salmonella* spp. can survive in foods with low *a_w_* values for periods as long as one year, the bacteria cannot grow until the moisture content of the product is increased [54]. This is the main reason why the product needs to be stored in a place with proper temperature and humidity conditions and protected from environmental contamination [69,70]. However, although *Salmonella* spp. present in the animal feed cannot grow, there is still a risk of contamination for the rodents. The infectious dose of *Salmonella* spp. is very low and can cause illness even though few viable cells are present [5].

In the case in which contamination of animal feed did occur, *Salmonella* spp. can survive in high numbers for extended periods [11,28]. In Figure 4, it is observed that *Salmonella* spp. population can persist in the animal feed during 64 days of storage at 17 °C. These results agree with several studies related to the survival of *Salmonella* spp. in foods with low moisture content. For example, Santillana and coworkers demonstrated that *Salmonella* spp. can survive in protein powder for 168 days at 21 °C, and it can survive in the food matrix even when the moisture content values are further decreased [11]. Another study conducted by Beuchat demonstrated that *Salmonella* spp. can survive in pecans stored at −20, 4, 21, and 37 °C for 546 days and 364 weeks in pecans chunks [68]. Hence, the ability of *Salmonella* spp. to survive for extended periods could represent a high risk of contamination for rodents, especially because the product is stored for at least one month before use.

### 4.4. UV and Thermal Resistance of Salmonella

*Salmonella* spp. inoculated in animal feed is not affected by UV light, which indicates that current inactivation protocol, applied by LEBi, is not serving the purpose of controlling microbial contamination of the animal feed. Similarly, other studies have reported no effect of UV light in food matrices with low *a_w_* content. For example, UV light is not adequate for the decontamination of spices in samples placed at a 10 cm distance from the light source; the *Salmonella* Typhimurium reduction was just 0.29 log CFU g^−1^. Another example is the minimal *Salmonella* spp. reduction reported in cumin seeds exposed to UV-C light during 60 min [71]. 

The low reduction in *Salmonella* from UV light could be attributed to the storage conditions of animal feed (used at LEBi) and the bacteria characteristics. The way the product is stored can affect the efficacy of the treatment as the animal feed is not directly exposed to the UV light and it is placed as far as 2 m from the primary source of radiation. It has been widely reported that bacterial inactivation is reduced when the product is far from the source of radiation and when the UV light is blocked [28,72]. Additionally, the animal feed is stacked vertically, which does not allow the radiation to be homogeneous throughout all the products. The way the animal feed is stacked does not allow the UV light to penetrate throughout the product, and the UV light cannot reach all the cells present [28,73]. This means that storage conditions do not follow the recommendations on the correct use of UV light. The UV light radiation may not affect *Salmonella* due to the protection mechanisms related to low *a_w_* environments [5]. It can be concluded that the disinfection mechanism with UV light does not have a significant effect on *Salmonella* spp. present in the product and that current ways of storing the animal feed could increase the risk of contamination for the rodents used for experimental analysis.

Given the limitations posed by UV light decontamination, additional intervention strategies for animal feed may be considered. This is important, especially in those cases where contamination occurs, and the possibility to obtain new batches of animal feed may be compromised. Thermal treatment of the product could be considered as an additional intervention strategy to control *Salmonella* as it could be easily applied in settings with limited resources, such as LEBi and other similar vivariums, in developing countries. Still, scientific literature shows that *Salmonella* spp. can survive well in food products with low *a_w_* after heating [11,74,75]. When exposed to low-*a_w_* environments, *Salmonella* spp. activates protein adaptation mechanisms that result in stable cell structures that decrease the thermal denaturation [76]. Other thermoresistance mechanisms include osmoregulation, ribosomal RNA degradation, filamentous shapes, biofilms formation, and activation of viable, but not culturable, bacteria. These mechanisms improve the resistance of *Salmonella* to heat, thus prolonging its survival in the food [74]. For example, Lound and coworkers reported that *Salmonella* Enteritidis increased its resistance 60 times more in dehydrated egg albumin than in nutrient broth at 72 °C and 300 times at 82 °C [74]. However, our study demonstrates that *Salmonella* spp. can be eliminated from the animal feed by treating samples at different time-temperature combinations [75]. The D-values obtained from this study are useful tools, and they are comparable to those reported in previous research. The D- values of *Salmonella* Senftenberg 775W in meat and bone flour with a moisture content of 10 g/100 g were 65.47, 37.37, 19.38, 6.60, 4.15, 2.08, 1.16, and 0.36 min at temperatures of 60, 63, 66, 71, 74, 77, 79, and 85 °C, respectively, with a *z*-value of 20.16 °C [15]. This type of data could be used to design proper intervention strategies for the decontamination of animal feed, and the *z*-values become practical tools to predict the behavior of *Salmonella* in this product. 

This study indicates that the current methodologies for storage and handling animal feed should be revised. New preservation methods such as heating the product (using an oven or autoclave) could be considered. However, although these recommended alternatives could help kill pathogenic bacteria, they could also affect the original nutritional composition of the product. Therefore, the application of new intervention strategies, for in-house applications, must be validated to prevent a deleterious effect on the product’s final quality [77].

## 5. Conclusions

Experimental animals may be at risk of contamination from the animal feed. Of special concern is the chronic consumption of mycotoxin-contaminated feed as this may undermine toxicological data (i.e., due to possible artifacts or confounding or undesired effects). 

Given the importance of vivariums, it is clear that keeping the well-being of the experimental animals should be in the priorities of different scientific units. This study provides input to generate recommendations to improve handling of the animal feed to prevent contamination issues and avoid compromising the quality and robustness of scientific studies. 

Monitoring of microbiological contamination could be improved by increasing the number of samples obtained per batch of animal feed. A robust sampling protocol could be applied considering the size of the batch and the distribution of the product within each sack. Additional testing should be incorporated to include the analysis of *Salmonella* and mycotoxins. Surveillance for pathogens, such as *Salmonella* spp. and mycotoxins, should be constant and rigorous. New data could be collected to determine the number of samples that must be necessary to assure proper monitoring of these variables. 

In terms of mycotoxins contamination, monitoring protocols could incorporate the analysis (with special emphasis on fumonisins and enniatins). 

Current strategy (e.g., UV light decontamination) is not serving its purpose and, according to our data, there is no use to maintain this handling protocol at the LEBi warehouse. Strengthening of monitoring protocols is the best way to compensate for the absence of any additional preventive measure. However, in case contamination occurs, D and z-values obtained from this study can be considered as an aid to design a post-processing decontamination protocol, especially in those cases where there is an urgent need of the animal feed and it is not possible to obtain additional product from the suppliers. Given the low water activity content of the product, heating protocols can be applied on product packaged in plastic bags suitable for autoclave use. This method is already applied by LEBi when sterilizing material that is sensitive to humidity. 

The information provided by this study, describes a reality that may be common to other countries where the access to adequate resources to perform experiments with animals could be limited. Therefore, other vivariums can use this information to take measures to improve the safety of the food they provide to their animals. Biomedical researchers may want to consider routinely testing feed for its quality and safety to, at least, foresee spurious effects caused by contaminants within the feed. 

## Figures and Tables

**Table 1:** Time-temperature combinations used to determine the thermal resistance of *Salmonella* spp.

Temperature, °C	Time, min					
65	0	30	60	90	120	150
70	0	20	40	60	80	-
75	0	15	30	45	60	-
80	0	6	12	18	24	-

**Table 2:** Descriptive analysis of the water activity (*a_w_*) values obtained for feed samples for laboratory animals.

Feed Supplier	Mean ± Standard Deviation *	Median	Maximum	Minimum
1	0.5120 ± 0.0993 ^a^	0.5103	0.6582	0.3846
2	0.5232 ± 0.0579 ^a^	0.5024	0.6183	0.4674

* Different letters indicate significant differences in concentration (*p* < 0.05).

**Table 3:** Descriptive and comparative analysis of mycotoxins carried out on different batches of balanced feed for laboratory animals from two different companies.

Toxin	Mean ± Standard Deviation *	Median	Maximum	Minimum
Concentration, μg kg^−1^				
Supplier 1				
15-ADON (*n* = 2/9, 22.2%)	398.45 ± 349.09	389.45	747.55	49.36
ZEA (*n* = 2)	92.55 ± 42.93	92.55	135.49	49.62
β-ZON (*n* = 3, 33.3%) ^a^	42.07 ± 6.76	43.39	49.62	33.22
FB_2_ (*n* = 3) ^a^	31.35 ± 10.80	23.95	46.62	23.48
FB_1_ (*n* = 4, 44.4%) ^a^	60.50 ± 26.53	69.78	84.71	17.74
ENNB_1_ (*n* = 4) ^a^	539.39 ± 333.37	612.27	917.47	15.56
AFB_1_ (*n* = 4)	3.30 ± 5.38	0.24	12.61	0.09
ENNB (*n* = 5, 55.5%) ^a^	1296.05 ± 1907.42	539.43	5090.18	110.50
Supplier 2				
DON (*n* = 2/7, 28.6%)	32.02 ± 2.89	32.02	34.91	29.13
HT-2 (*n* = 2)	28.64 ± 17.02	28.64	45.67	11.62
OTA (*n* = 2)	84.90 ± 8.66	84.90	93.56	76.24
STE (*n* = 2)	36.00 ± 22.52	36.00	58.52	13.47
ZEA (*n* = 2)	6.80 ± 2.69	6.80	9.50	4.10
AFG_2_ (*n* = 3, 43.8%)	7.47 ± 10.53	0.043	22.37	0.022
ENNB_1_ (*n* = 4, 57.1%) ^a^	167.45 ± 136.82	160.00	330.14	19.66
ENNB (*n* = 6, 85.7%) ^a^	225.65 ± 156.30	233.89	503.64	25.44
β-ZON (*n* = 7, 100%) ^b^	268.71 ± 527.85	78.62	1559.62	10.08
FB_1_ (*n* = 7)	958.22 ± 2080.44	29.99	6044.04	0.76
FB_2_ (*n* = 7)	147.37 ± 147.94	64.05	421.53	0.54

* Toxins with superscripted letters are compared among suppliers. Different letters indicate significant differences in concentration (*p* < 0.05).

**Table 4:** Descriptive analysis of toxins found over time in two batches of animal feed stored in the laboratory.

Toxin *	Mean ± Standard Deviation ^§^	Median	Maximum	Minimum
Concentration, μg kg^−1^				
Supplier 1, sample S1I				
AFG_2_ (*n* = 4)	0.41 ± 0.48	0.16	1.35	0.05
ENNB_1_ (*n* = 5)	157.47 ± 97.16	111.01	329.25	58.81
ENNB (*n* = 6)	279.46 ± 230.49	211.64	778.65	64.77
β-ZON (*n* = 6)	157.71 ± 93.95	144.58	328.01	13.75
FB_2_ (*n* = 7)	14.85 ± 8.27	11.53	31.11	6.08
FB_1_ (*n* = 9)	159.90 ± 137.44	94.53	380.93	14.67
Supplier 2, sample S2A				
AFG_2_ (*n* = 4)	0.39 ± 0.50	0.14	1.24	0.03
DON (*n* = 4)	50.21 ± 42.33	28.79	123.92	22.00
ENNB (*n* = 7)	331.22 ± 177.41	335.98	693.98	50.24
ENNB_1_ (*n* = 5)	83.19 ± 62.49	72.05	200.01	25.80
FB_1_ (*n* = 9)	263.14 ± 132.46	235.79	446.80	86.34
FB_2_ (*n* = 9)	24.33 ± 9.03	24.61	38.34	10.96
STE (*n* = 3)	145.97 ± 180.50	29.11	400.94	7.86
β-ZON (*n* = 5)	156.58 ± 154.08	94.75	462.12	41.71

*, ^§^ Values in brackets represent the frequency in which each toxin was found in the food batches. Descriptive data expressed as toxin prevalence, i.e., contemplates only analytes with values above detection limits [10].

**Table 5:** Decimal reduction times (D-values) obtained from the thermal destruction of *Salmonella* spp. in animal feed treated at different temperatures.

Temperature, °C	D-Value ± Standard Deviation, min	log (D-Value) ± Standard Deviation, min
65	65.58 ± 2.95	1.8101 ± 0.0198
70	23.53 ± 0.39	1.3717 ± 0.0072
75	15.4 ± 21.00	1.1881 ± 0.2830
80	6.21 ± 0.11	0.7931 ± 0.0079

## Data Availability

Not applicable.
